# Changes in creatine transporter function during cardiac maturation in the rat

**DOI:** 10.1186/1471-213X-10-70

**Published:** 2010-06-22

**Authors:** Alexandra Fischer, Michiel ten Hove, Liam Sebag-Montefiore, Helga Wagner, Kieran Clarke, Hugh Watkins, Craig A Lygate, Stefan Neubauer

**Affiliations:** 1 Department of Cardiovascular Medicine, Wellcome Trust Centre for Human Genetics, University of Oxford, Roosevelt Drive, Oxford, UK; 2 Department of Cardiology, Medizinische Universitätsklinik Würzburg, 97080 Würzburg, Germany; 3 Department of Physiology, University of Oxford, South Parks Road, Oxford, UK

## Abstract

**Background:**

It is well established that the immature myocardium preferentially utilises non-oxidative energy-generating pathways. It exhibits low energy-transfer capacity via the creatine kinase (CK) shuttle, reflected in phosphocreatine (PCr), total creatine and CK levels that are much lower than those of adult myocardium. The mechanisms leading to gradually increasing energy transfer capacity during maturation are poorly understood. Creatine is not synthesised in the heart, but taken up exclusively by the action of the creatine transporter protein (CrT). To determine whether this transporter is ontogenically regulated, the present study serially examined CrT gene expression pattern, together with creatine uptake kinetics and resulting myocardial creatine levels, in rats over the first 80 days of age.

**Results:**

Rats were studied during the late prenatal period (-2 days before birth) and 7, 13, 21, 33, 50 and 80 days after birth. Activity of cardiac citrate synthase, creatine kinase and its isoenzymes as well as lactate dehydrogenase (LDH) and its isoenzymes demonstrated the well-described shift from anaerobic towards aerobic metabolism. mRNA levels of CrT in the foetal rat hearts, as determined by real-time PCR, were about 30% of the mRNA levels in the adult rat heart and gradually increased during development. Creatine uptake in isolated perfused rat hearts increased significantly from 3.0 nmol/min/gww at 13 days old to 4.9 nmol/min/gww in 80 day old rats. Accordingly, total creatine content in hearts, measured by HPLC, increased steadily during maturation (30 nmol/mg protein (-2 days) vs 87 nmol/mg protein (80 days)), and correlated closely with CrT gene expression.

**Conclusions:**

The maturation-dependant alterations of CK and LDH isoenzyme activities and of mitochondrial oxidative capacity were paralleled by a progressive increase of CrT expression, creatine uptake kinetics and creatine content in the heart.

## Background

Postnatal maturation of the heart is associated with substantial changes in energy metabolism, one of the key determinants of performance. Foetal and newborn hearts are relatively more dependent on anaerobic glycolysis, using mostly glucose, whereas the mature heart is almost exclusively aerobic, with free fatty acids as the predominant substrate [1,2]. The transition from carbohydrate to fatty acid metabolism (i.e. anaerobic to aerobic metabolism) is well documented [3,4] and involves processes, such as maturation of mitochondria and changes in circulating levels of fatty acids and lactate [2]. Thus, the immature myocardium is characterised by lower mitochondrial content [5] and lower activities of both tricarboxylic acid cycle and electron transport chain enzymes [6,7]. Furthermore, contents of both phosphocreatine and total creatine [8], as well as creatine kinase activity [9-11] show a monotonic increase during cardiac maturation. Specifically, myocardial MM-CK and mito-CK isoenzymes increase [12-15], while relative activities of BB-CK and MB-CK isoenzymes decrease during maturation [16,17].

Creatine is not synthesised in the heart, but taken up by cardiomyocytes exclusively by action of the creatine transporter (CrT), a 55 Kda-plasma membrane protein [18]. This transporter is likely to be involved in the regulation of creatine content in the cardiomyocyte during maturation. One recent report focused on regulation of creatine metabolism during pregnancy in spiny mice [19]. However, to date, no studies have reported on CrT expression and function during cardiac maturation in rats, and in particular, spanning from in utero, through adolescence, and into adulthood.

Therefore, the present study was designed to serially examine the mechanisms by which maturation affects transcription and function of CrT in the heart. For this purpose, the CrT mRNA levels were measured in rats of different age using real-time RT-PCR. All currently available anti-CrT antibodies cross-react with non-CrT polypeptides [20] and consequently a reliable evaluation of CrT protein expression is currently not feasible. Therefore, creatine transporter function in the developing rat myocardium was assessed by measuring creatine uptake by means of perfusing hearts with ^14^C-creatine.

## Results

### Heart weight, body weight and cardiac function

Table 1 shows characteristics of rats during 7 stages of development. During perinatal development, as expected, there was a progressive increase of heart weight (HW) and body weight (BW). The heart weight/body weight ratio decreased significantly from 10.9 ± 2.1 mg heart/g body weight in fetuses to 3.94 ± 0.1 mg heart/g body weight in adults (p < 0.001). Heart rate (HR), obtained in perfused heart experiments (13 days old onwards) was unchanged over time. Left ventricular developed pressure (LVDP) was not significantly different in isolated, buffer-perfused hearts.

**Table 1:** Heart weights (HW), body weights (BW), cardiac function, plasma creatine content and creatine uptake during development in the rat heart

Age (days)	-2 ( fetal )	7	13	21	33	50	80	ANOVA
**HW (g)**	0.039 ± 0.01^a^	0.13 ± 0.01^b^	0.17 ± 0.06^bc^	0.24 ± 0.03^c^	0.44 ± 0.04^d^	0.70 ± 0.07^e^	1.17 ± 0.02^f^	**p < 0.001**
	[4]	[4]	[7]	[5]	[8]	[6]	[8]	
**BW (g)**	3.59 ± 0.34^a^	22.0 ± 1.78^b^	35.6 ± 7.08^c^	41.5 ± 4.1^c^	78.6 ± 2.4^d^	178 ± 8.6^e^	296 ± 5.3^f^	**p < 0.001**
	[4]	[4]	[7]	[5]	[8]	[6]	[8]	
HW/BW *1000	10.9 ± 2.1^a^	5.79 ± 0.80^ab^	4.81 ± 1.15^ab^	5.84 ± 0.70^a^	5.58 ± 0.47^a^	3.93 ± 0.32^b^	b 3.94 ± 0.10^b^	**p < 0.001**
	[4]	[4]	[7]	[5]	[5]	[6]	[8]	
**HR **(bpm)	-	-	321 ± 5.7	248 ± 4.9	348 ± 5.6	318 ± 34	307 ± 45	**NS**
			[4]	[4]	[4]	[4]	[4]	
**LDVP **(mm Hg)	-	-	69 ± 12.7	88.6 ± 0.36	65.7 ± 7.2	75.7 ± 10.2	97.1 ± 27.6	**NS**
			[4]	[4]	[4]	[4]	[4]	
**RPP **(mm Hg/bpm)	-	-	22129 ± 3673	22047 ± 555	22879 ± 2874	24189 ± 5524	29673 ± 8913	**NS**
			[4]	[4]	[4]	[4]	[4]	
**Creatine uptake**	-	-	3.03 ± 0.55^a^	3.66 ± 0.79^ab^	3.85 ± 0.62^abc^	4.68 ± 0.54^bc^	4.99 ± 0.52^c^	**p < 0.001**
(nmol/min/g w.w)			[6]	[5]	[5]	[5]	[6]	
**Plasma creatine**	-	0.971 ± 0.07^a^	0.669 ± 0.16^ab^	0.678 ± 0.19^ac^	0.627 ± 0.12^bc^	0.558 ± 0.06^bc^	0.536 ± 0.08^bc^	**p = 0.002**
(mmol/L)		[4]	[4]	[4]	[4]	[4]	[4]	

Data are presented as means ± S.E and sample size are given in brackets. Values within a row with different superscript letters are significantly different to all other time points (p < 0.05, creatine uptake, plasma creatine: Bonferroni; HW, BW, HW/BW*1000: Dunnett T3, ANOVA "test for linear trend": as indicated).

NS = not significant, p > 0.05

### Plasma creatine levels, myocardial creatine content and cardiac enzyme activities

The mean plasma creatine concentrations, which could be obtained from 7 day old animals onwards, are also shown in Table 1. During maturation there was a steady decrease of up to 44% in plasma creatine concentration (0.97 ± 0.07 mmol/L (7 days) vs 0.54 ± 0.08 mmol/L (80 days), reaching significance (p < 0.05) from 33 days of age onwards.

**Figure 1:**
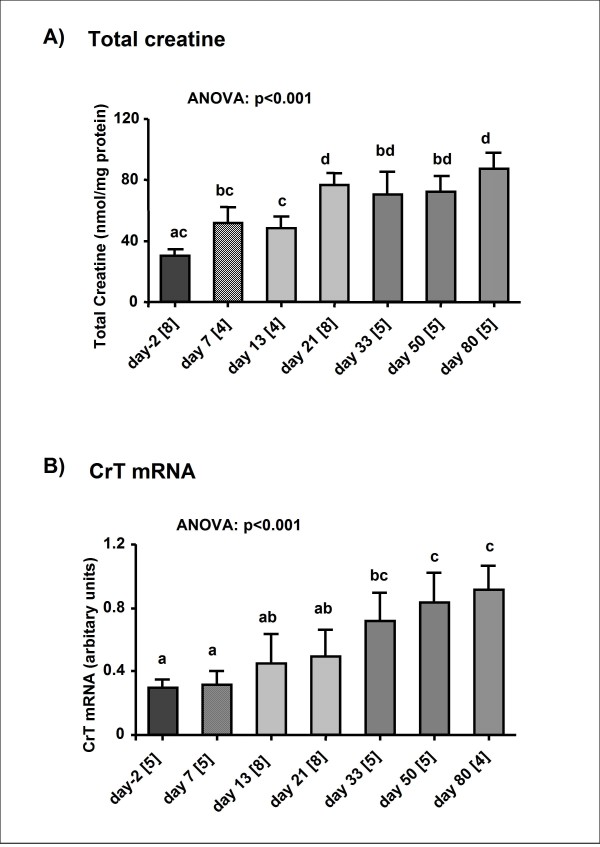
**Total creatine content and creatine transporter mRNA in rat heart during maturation**. **A)**. Myocardial total creatine content in rats aged -2, 7, 13, 21, 33, 50 and 80 day old. **B) **Creatine transporter (CrT) mRNA levels in rat heart during maturation. Total RNA was isolated from foetal hearts on day 20 of gestation (2 d before birth), and 7, 21, 33, 50 and 80 d after birth. The level of CrT mRNA was measured by real-time RT-PCR and normalized to β-actin. Values are mean ± S.E. and sample size are given in brackets on the labels of the X axis. Data points not sharing the same superscript letter indicate total creatine and mRNA level respectively are significantly different to all others (p < 0.05 Bonferroni, ANOVA "test for linear trend": p < 0.001).

**Figure 2:**
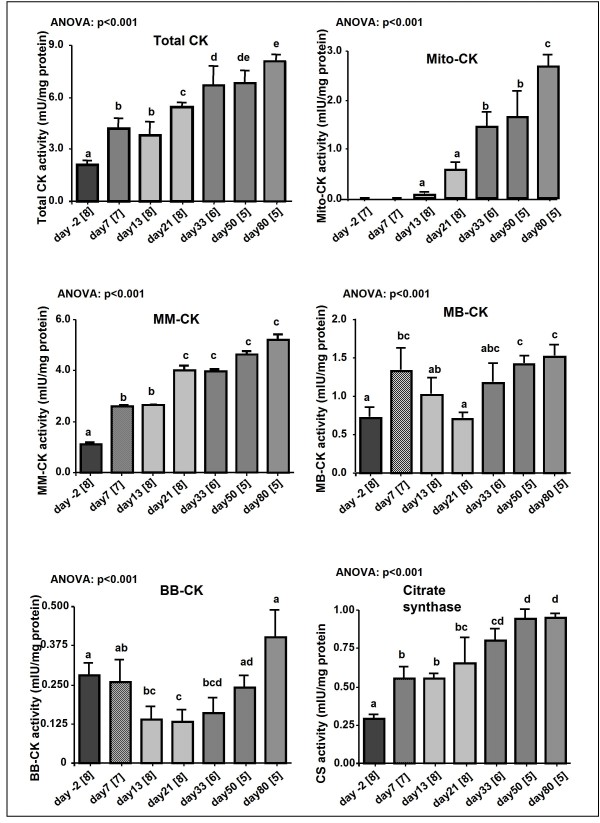
**Total creatine kinase (CK) and its isoenzyme activities and citrate synthase (CS) activity during maturation**. Values are means ± S.E. and sample size are given in brackets on the labels of the X axis. Data points with different superscript letters are significantly different to all other timepoints (p < 0.05, CK, CS: Bonferroni; Mito-CK, MM-CK, MB-CK, BB-CK: Dunnett T3, ANOVA "test for linear trend": as indicated).

Total creatine content (Figure 1A) in the heart significantly (ANOVA p < 0.001) increased during maturation from 30.1 ± 1.3 nmol/mg protein in -2 day old rats to 87 ± 2.9 nmol/mg protein in 80 day old rats, corresponding to a ~3-fold increase. Similarly, total creatine kinase (CK) activity increased several-fold (p < 0.001) during maturation (2.1 ± 0.26 mIU/mg protein (-2 days) vs 8.06 ± 0.4 mIU/mg protein (80 days) (Figure 2)). Compared with adult heart, B-containing CK isoenzymes are relatively more abundant in foetal myocardium, but total CK activity is much lower. Calculating CK isoform activities from total CK and % isoenzyme distribution revealed that the BB isoform did not increase until 80 days of age (ANOVA: p = 0.01), whilst MB- (2.1-fold, p < 0.001) and MM-isoforms (4.7-fold, p < 0.001) increased directly after birth. The activity of mito-CK, which was not detectable until at 13 days after birth, increased during growth from 0.0075 ± 0.05 to 2.68 ± 0.26 mIU/mg protein. Citrate synthase activity, a marker of mitochondrial volume, rose significantly (p < 0.001) in a steady manner throughout the perinatal period and reached maximum levels in the adult of 0.95 ± 0.03 mIU/mg protein.

**Figure 3:**
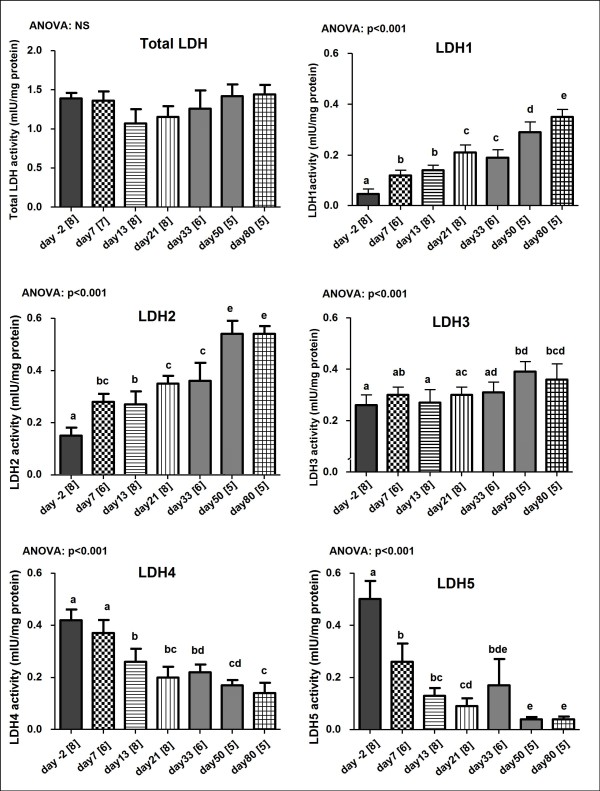
**Total lactate dehydrogenase (LDH) and its isoenzyme activities during maturation**. Values are means ± S.E. and sample size are given in brackets on the labels of the X axis. Data points with different superscript letters are significantly different to all other timepoints (p < 0.05, LDH1, LDH2, LDH3, LDH4: Bonferroni; LDH5: Dunnett T3, ANOVA "test for linear trend": as indicated). Note: NS = not significant, p > 0.05

Figure 3 shows the developmental pattern of total lactate dehydrogenase (LDH) activity. Although no significant difference was found in total LDH activity during maturation, the distribution of LDH isoenzyme activities changed significantly. LDH_1_, LDH_2 _and LDH_3 _isoenzymes showed a significant increase (~7.4, ~3.6 and 1.4 fold respectively), while LDH_4 _and LDH_5 _isoenzymes decreased (~3 and 12.5 fold respectively). This shift in LDH isoenzyme activity reflects the change from anaerobic to aerobic metabolism during maturation.

### Creatine transporter gene expression

To explore changes in CrT mRNA expression during maturation, the total RNA isolated from rat hearts of different age groups was analyzed using a real-time quantitative RT-PCR technique. The abundance of the transcripts for CrT increased steadily with age (Figure 1B). At 80 days of age, cardiac mRNA levels of CrT were significantly higher compared with all other experimental groups, showing a ~3.6 fold higher relative expression of CrT as compared to -2 day old rats (ANOVA p < 0.001).

### Creatine uptake

CrT function was examined by measuring creatine uptake by means of ^14^C creatine labeled heart perfusion. Table 1 shows the rate of creatine uptake at 500 μmol/L extracellular creatine in 13 to 80 day old rats. Creatine uptake steadily increased with age and was significantly higher than in 13 day old hearts from day 50 onwards. Creatine uptake was 3.0 ± 0.6 nmol/min/gww in the 13 day old heart and reached 4.9 ± 0.5 nmol/min/gww at 80 days of age, i.e. increased by ~65%. Thus, creatine content, CrT expression and CrT function all increased steadily during maturation.

### Correlations of creatine content and uptake with gene expression

**Figure 4:**
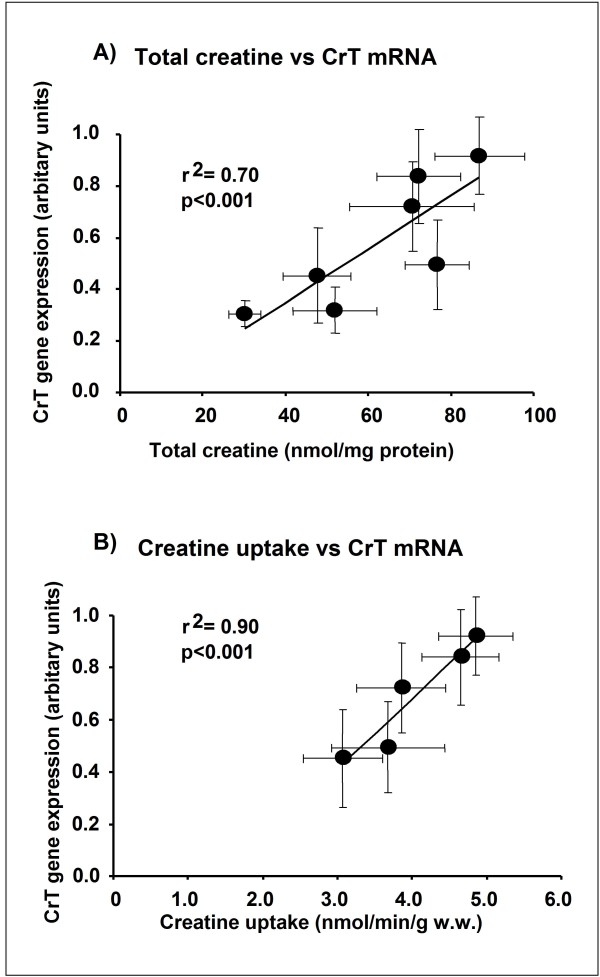
**Relationship between CrT mRNA and total creatine (A) and between CrT mRNA and creatine uptake (B), indicated by Pearson's correlation coefficient**. Each data point (mean ± S.E.) represents a different developmental age from -2, 7, 13, 21, 33, 50 and 80 days post-partum. CrT mRNA and total creatine data were collected from the same hearts, whereas creatine uptake data was collected from different hearts. There are no data points in (B) for days -2 and 7 since it was not possible to measure creatine uptake in these very small hearts.

There was a significant correlation (r^2 ^= 0.70, p < 0.001) between CrT mRNA and total creatine levels (Figure 4A) as well as CrT mRNA and creatine uptake (r^2 ^= 0.90, p < 0.001, Figure 4B).

## Discussion

To the best of our knowledge, this is the first study to examine changes in myocardial creatine transporter (CrT) mRNA levels, creatine uptake and content during cardiac maturation, which includes the pre-natal period (-2 d), early postnatal period (7 d, 13 d), the immediate post-weaning period (21 d, 33 d), and the period of sexual maturity (50 d) and adult life (80 d).

### Changes of substrate utilisation and myocardial energetics during maturation

The velocity of the CK reaction changes with the size of the creatine pool and also with the accumulation of CK isoenzymes and the shift in isoenzyme distribution [14]. The appearance of CK function in myofibrils and mitochondria during maturation has been studied in a number of animal models [16,12,9,10]. In agreement with previous studies [13,14], we showed that mito-CK, bound on the inner membrane of the mitochondria, and MM-CK, a significant portion localised at sites of ATP utilisation, e.g. myofibrils, are the predominant isoenzymes in mature myocardium, whereas BB-CK and MB-CK isoenzymes dominate in the immature myocardium. Adult levels of mito-CK are not reached until late in development (50 days), suggesting that its role in mediating PCr synthesis from oxidative phosphorylation is required at a relatively late stage of maturation. These findings confirm previous results and show that the maturational changes in creatine metabolism described occur in parallel to the expected changes in the creatine kinase system.

In line with previous studies [17], total creatine content in hearts increased 3-fold during maturation. Recent findings from Ireland et al [19] in the spiny mouse, which, unlike conventional rats and mice, has a long gestation of 38-40 days, revealed that the estimated amount of total creatine did not change in the foetal heart between 30-37 days gestation, but it increased significantly by postnatal day 10. In the fetus, myocardial creatine is derived both from the mother and from *de novo *synthesis. During maturation myocardial creatine levels increase as the demand for all energy-requiring processes in the heart rises. Likewise, myofibrillar ATPase activity increases steadily during the perinatal period [12]. Thus, energy producing and energy utilising reactions are concomitantly increased within the cardiomyocyte.

### Alterations of LDH isoform expression and citrate synthase activity

In our study, changes in total LDH activity were accompanied by distinct changes in LDH isoenzyme pattern. Specifically, we observed an increase in LDH_1_, LDH_2 _and LDH_3 _isoenzymes. LDH_1 _and LDH_2 _were predominant in the adult heart, whilst LDH_4 _and LDH_5 _were found to dominate in the foetal heart. These changes are again in line with previous findings [21] and are indicative of the shift from anaerobic to aerobic metabolism during maturation.

Similarly, citrate synthase, a marker of mitochondrial volume, increased during maturation. A rise in mitochondrial numbers is consistent with previous studies [22] and reflects the increased energy demand of mature myocardium.

### Regulation of creatine uptake and creatine transporter expression during maturation

Studies using cultured cells [23-25], isolated rat hearts [26] and *in vivo *human studies [27] indicate that muscle cells tightly regulate their intracellular creatine content. However, the underlying cellular and molecular mechanisms by which these cells perform this task are only partially understood. The two enzymes required for creatine biosynthesis L-arginine:glycine amidinotransferase and S-adenosylmethionine:N-guanidinoacetate methyltransferase [28] may be expressed in heart tissue [29], but activities of these enzymes are negligible and therefore creatine is not synthetised in heart muscle in significant amounts. Instead, creatine is taken up into the heart by the action of the creatine transporter. During maturation, cardiac creatine levels increase steadily. Therefore, we hypothesized that during development the CrT-governed creatine uptake is progressively increased.

Indeed, we found the maximal rate of creatine transport to steadily increase during maturation, in parallel with both total creatine content and creatine transporter mRNA levels. Importantly, we found a significant correlation (r^2 ^= 0.70) between CrT mRNA and total creatine levels as well as CrT mRNA and creatine uptake (r^2 ^= 0.90). Thus during development, 90% of the total variance in creatine uptake capacity and 70% of the variance in creatine content could be explained by changes in gene expression of the CrT. This is in contrast to the adult heart, where changes in CrT activity in response to genetically altered creatine content were mainly driven by non-transcriptional means, e.g. substrate feedback or post-translational modification [30].

The near maximum rate for ^14^C-creatine uptake in isolated perfused 80 day old rat hearts (4.99 ± 0.52 nmol/min/gww) agreed with previously published data on adult rat heart [31,26,32]. The observed increase in heart weight does not explain the observed maturation-induced increase in Na^+^/Cl^- ^creatine transport activity because the uptake values were normalised to mg of protein. Likewise, Garcia-Delgado et al [33] found an up-regulation of renal Na^+^/Cl^- ^creatine transporter during ontogeny in rats. In their study, age increased V_max_, indicating that renal maturation may have increased the density of creatine transporters and/or their turnover rate.

Similar to our findings, Braissant et al [34] showed detectable CrT mRNA levels in the rat embryo at 18.5 day of gestation, while at day 12.5 and 15.5, mRNA levels were absent. Ireland et al [19] recently demonstrated in the spiny mouse that, between gestational day 37 and postnatal day 10, cardiac mass increased almost 3-fold and total creatine content increased 2-fold. CrT mRNA expression, however, did not increase significantly between mid-gestation and gestational day 37 and stayed at similar levels until postnatal day 10. Thus, the authors suggest that creatine uptake into the heart is facilitated by an increase in CrT activity rather than CrT protein expression. In contrast, we found a significant increase of CrT mRNA expression in the rat heart at day 13 compared to pre-gestation (-2 days).

The reason for decreasing plasma concentration of Cr at different ages may be related to the fact that Cr accumulates in several organs and tissues (e.g. heart, brain [35] and skeletal muscle [36] at later stages of development, likely trough an increase in Cr uptake. Higher uptake may in turn lead to lower Cr levels in the plasma as observed in the present study. In fact, for the heart we have found an inverse relationship (r^2 ^= 0.588, p < 0.05) between plasma Cr and CrT gene expression. A decrease in intestinal Cr uptake during development [37] may add to this phenomenon of decreasing plasma concentration.

Furthermore, phosphorylation of CrT by cAMP-dependent protein kinase A and protein kinase C, which affect CrT activity, may be an underlying signal transduction pathway by which the CrT in heart is regulated during early development after birth as suggested by others [37].

### Limitations

Unfortunately, to date it remains impossible to determine how the increases in CrT mRNA, activity and creatine content are paralleled by an increase in CrT protein content, since all available anti-CrT antibodies cross-react with pyruvate dehydrogenase and other proteins [20]. Therefore, determining the changes in CrT protein expression during development including post-translational regulatory mechanisms remains an important milestone for the future. Another limitation of our study is that we did not perform measurements of high-energy phosphate metabolites ATP, phosphocreatine, and of the free energy change of ATP hydrolysis (delta-G). However, on wet chemical analysis, phosphocreatine is highly unstable and it would be a challenge to measure this reliably in the smallest hearts. Furthermore, delta-G requires measurement of intracellular pH by ^31^P-MRS in isolated perfused hearts, which is currently not technically possible in the youngest age groups. These analyses are important goals for future research.

## Conclusions

Our work shows that the typical alterations of CK and LDH isoenzymes and of mitochondrial oxidative capacity observed during heart maturation are paralleled by a progressive increase of creatine uptake kinetics. Moreover, CrT mRNA in rat heart increased steadily during maturation and this increase was reflected by the myocardial creatine content. Our data suggest that the age-dependent increase in the activity of the PCr/CK system is closely paralleled by increases in CrT mRNA expression, Cr uptake and cardiac Cr content, reflecting the increasing requirement of the heart for ATP turnover over the first 80 days of life.

## Methods

### Experimental animals

All experimental procedures in this study were approved by the University of Oxford Animal Ethics Review Committees and by the Home Office (London, U.K). Rats used in all studies were of the Wistar strain. Timed pregnant rats were obtained from a commercial breeder (Harlan, U.K) and kept under controlled conditions for temperature, humidity and light, with creatine-free rat chow and water available *ad libitum*. Pups were weaned at 21 days and placed on the same chow as the mother. Fetuses at 18 days after gestation (-2 days) were obtained by Caesarean section after anesthetising the mother with 1.5-2% isoflurane. Animals of age 7, 13, 21, 33, 50 and 80 days were used.

### Heart preparation

Animals were anesthetized with an intraperitoneal injection of 75 mg/kg body wt sodium pentobarbitone (Sagatal, Rhône Mérieux, Dublin), whilst foetuses were sacrificed by cervical dislocation. After loss of pedal reflexes, blood samples were removed from the femoral vein (animals aged 7 days onwards) and hearts were rapidly excised and arrested in ice-cold Krebs-Henseleit buffer (see below). Hearts from 13, 21, 33, 50 and 80 day old rats were cannulated via the ascending aorta for retrograde perfusion radiolabelled ^14^C-creatine transport studies and metabolite assays (-2 and 7 day old hearts were too small for perfusion). A water-filled cling film balloon, attached via polytetrafluorethylene tubing to a Gould disposable pressure transducer, was inserted into the left ventricular cavity via the mitral valve, secured by a ligature and inflated sufficiently to give an end-diastolic pressure (EDP) of ~4 mm Hg. Heart rate and left ventricular (LV) pressures were recorded continuously using a MacLab 4a (AD Instruments Ltd, Hastings, UK) linked to Chart™ software (AD Instruments).

### Radioactive creatine uptake assays

Hearts from > 13 days old rats (different from the rats used for the biochemical analysis, which in our setup is not possible on radioactive samples) were perfused with Krebs-Henseleit (KH) buffer containing (mmol/L) NaCl (110), KCl (4.7), MgSO_4_.7H_2_O (1.2), CaCl_2_.2H_2_O (1.75), glucose (11), KH_2_PO_4 _(2), NaHCO_3 _(25), Na_2_EDTA (0.5), Na-Lactate (0.5) and Na-Pyruvate (4.5). The buffer, supplemented with 500 μmol/L creatine, was gassed with 95% O_2_/5% CO_2 _to give a pH of 7.4 at 37°C. Creatine concentration was used at the optimal level of 500 μmol/L, since Cr uptake by adult rat hearts at this concentration, approaches Vmax [26,31]. Hearts were perfused with a recirculating volume of ca. 400 ml at a constant pressure of 100 mmHg. Cardiac contractile function was monitored throughout the protocol as described above. Since the accumulation of ^14^C-labelled creatine in the heart has been shown to be linear throughout a 1 hr incubation [26], 30 minutes was used as the optimal radioactive label perfusion time. Hearts were allowed to equilibrate for 10 min and then ^14^C-labelled creatine (Apin Chemicals Ltd, Abingdon, U.K) was added in trace amounts to the recirculating buffer. Hearts were perfused in the presence of ^14^C-creatine for 30 minutes, after which time a final 15 min label washout period followed, using a non-circulating mode with non-radioactive buffer. The absence of ^14^C-label in the effluent between 10 and 15 min of washout ensured that the extracellular space had been cleared and that creatine leakage from the heart was negligible. At the end of the perfusion protocol, hearts were weighed and scar tissue removed. Subsequently, hearts were digested in 1 ml of 1 M KOH at 60°C before scintillation counting for [^14^C] as described previously [26].

### RNA isolation and creatine transporter mRNA expression

Total RNA was extracted from frozen heart tissue using the RNeasy Kit (Qiagen), including treatment with proteinase K according to manufacturer's instructions. Total tissue RNA dissolved in RNase-free water was used in real-time RT-PCR (Qiagen Quantitect SYBR Green RTPCR kit, Qiagen, Crawley, United Kingdom), along with the following primers: RT-CrT-F 5'-gccggcagcatcaatgtc-3', RT-CrT-R 5'-ggtgttgcagtagaagacgatcac-3'. Quantitative fluorescent real-time RT-PCR analysis was performed to investigate mRNA expression levels in rat hearts of different age using the Rotor-Gene system (Corbett Research Ltd., Cambridge, United Kingdom). To demonstrate linearity of the reactions, a standard curve was generated using a series of total RNA dilutions from 80-day-old rat hearts. Results were normalized to the expression levels of the housekeeping gene β-actin (RTactinF 5'-gacaggatgcagaaggagattact-3', RTactinR 5'-tgatccacatctgctggaaggt-3'). Real-time RT-PCR was performed in duplicate with 8-10 ng of total RNA as input. Quantification was carried out using appropriate software to generate standard curves, which expressed relative quantities of PCR products in the experimental samples in arbitrary units relative to the standard curve.

### Plasma preparation

Total blood samples were centrifuged at 3,000 rpm for 10 min. The supernatant was used for the determination of plasma concentrations of creatine by HPLC as previously described [38].

### Myocardial enzymes and creatine measurements

Hearts were perfused for 10 minutes before being rapidly freeze-clamped using Wollenberger tongs. Aliquots were taken to measure protein content by the method of Lowry [39]. 0.1% Triton X was added and enzyme activities of citrate synthase, CK and LDH were measured using an Ultraspec 2100 pro (Pharmacia Biosystems) as previously described [37]. CK isoenzyme distribution was measured using the Rapid Electrophoresis System (REP, Helena Biosciences, UK) as a separation unit, and the REP CK isoforms kit (Helena Biosciences, UK). For measurements of LDH isoenzymes, the Titan Gel LD Isoenzyme System (REP, Helena Biosciences, UK) was used. The Electrophoresis Data Center (EDC, Helena Biosciences, UK) automatically quantified the separated isoenzyme band. For HPLC analysis, tissue was homogenised in 0.4 N perchloric acid at 4°C, neutralised and centrifuged for 5 min at maximum speed, at 4°C. The supernatant was used to measure total creatine levels as previously described [40]. Tissue concentrations were expressed in nmol/mg of protein.

### Statistical analysis

Data are expressed as the mean ± SE and sample size is given in brackets. To determine statistical significance between age groups, test for linear trend in one way analysis of variance (ANOVA) was performed, followed by a Bonferroni multiple comparison test. Every time point was compared with every other time point. In case of inhomogeneity of variance the Dunnet T3 test was used. P values less than 0.05 were considered significant. Correlation analysis was conducted for selected variables. All statistical analyses were performed by SPSS (Version 13.0).

## Authors' contributions

AF carried out all animal studies, the analysis of CrT mRNA and drafting of the manuscript, MTH carried out the creatine uptake assays and LS-M and HW performed the myocardial enzymes and creatine measurements, KC and HW assisted the experimental development and CAL and SN were responsible for the concept and design of the study and the drafting of the paper. All authors read and critically revised the final manuscript.
